# Immobilization of *Aspergillus niger* F7-02 Lipase in Polysaccharide Hydrogel Beads of *Irvingia gabonensis* Matrix

**DOI:** 10.1155/2014/967056

**Published:** 2014-12-31

**Authors:** Safaradeen Olateju Kareem, Olayinka Quadri Adio, Michael Bamitale Osho

**Affiliations:** ^1^Department of Microbiology, Federal University of Agriculture, PMB 2240, Abeokuta 110001, Ogun State, Nigeria; ^2^Department of Biological Sciences, McPherson University, Seriki-Sotayo, PMB 2094, Abeokuta 110116, Ogun State, Nigeria

## Abstract

The potential of polysaccharide *Irvingia gabonensis* matrix as enzyme immobilization support was investigated. Lipase of *Aspergillus niger* F7-02 was immobilized by entrapment using glutaraldehyde as the cross-linking agent and stabilized in ethanolic-formaldehyde solution. The pH and temperature stability and activity yield of the immobilized enzyme were determined. Such parameters as enzyme load, bead size, number of beads, and bead reusability were also optimized. Adequate gel strength to form stabilized beads was achieved at 15.52% (w/v) *Irvingia gabonensis* powder, 15% (v/v) partially purified lipase, 2.5% (v/v) glutaraldehyde, and 3 : 1 (v/v) ethanolic-formaldehyde solution. There was 3.93-fold purification when the crude enzyme was partially purified in two-step purification using Imarsil and activated charcoal. Optimum lipase activity 75.3 Ug^−1^ was achieved in 50 mL test solution containing 15 beads of 7 mm bead size. Relative activity 80% was retained at eight repeated cycles. The immobilization process gave activity yield of 59.1% with specific activity of 12.3 Umg^−1^ and stabilized at optimum pH 4.5 and temperature 55°C. Thus the effectiveness and cost-efficiency of *I. gabonensis* as a polymer matrix for lipase immobilization have been established.

## 1. Introduction

Lipases (glycerol ester hydrolases, E.C. 3.1.1.3) are hydrolytic enzymes that act in aqueous-organic interfaces, catalysing the cleavage of ester bonds in triglycerides and producing glycerol and free fatty acids [1]. The process of lipid modification using lipases is currently attracting great interest. The reasons for this enzymes great biotechnological potential, besides the different types of reaction that they can catalyze, include their high stability in the presence of organic solvents, the lack of need for cofactors, and their ability to catalyze reactions with chemo-, regio-, and enantioselectivity [2]. The use of enzymes and other proteins has been limited due to their considerably unstable nature and the resulting requirement of stringent conditions, such as a particular pH and temperature. Several techniques have therefore been studied and utilized to overcome these problems and optimize various applications. In chemical and biochemical reactions, purified enzymes can be rather costly and to discard them after each use is not economical. Retrieval of enzymes from the reaction medium, however, can be difficult and therefore researchers have explored the immobilization of enzymes [3]. Greater stability and enzyme activity are typically exhibited over a broader range of pH and temperature by immobilized enzymes [4]. The immobilization of enzyme on insoluble supports has been a topic of active research in enzyme technology and is essential for their application to individual processes, for example, in food technology, biotechnology, biomedicine, and analytical chemistry [5]. For industrial application, the immobilized form of enzyme offers several advantages, including repeated use of the enzyme, ease of product separation, improvement of enzyme stability, and continuous operation in packed-bed reactors [6]. Immobilization by entrapment differs from other techniques such as adsorption and covalent binding in that enzyme molecules are free in solution, but restricted in movement by the lattice structure of a gel [7]. The porosity of the gel lattice is controlled to ensure that the structure is tight enough to prevent leakage of enzyme or cells yet at the same time allows free movement of substrate and product. The support also acts as a barrier and can be advantageous as it protects the immobilized enzyme from microbial contamination by harmful cells, proteins, and enzymes in the microenvironment [8]. Different polysaccharide hydrogel beads have been used as supports for lipase entrapment immobilization. This includes agarose beads, alginate beads [3], chitosan beads [9], k-carrageenan beads [10], and nanogel beads [11]. Osho et al. [12] have reported the use of bionatural matrices—structural fibrous network (SFN) and vegetable sponge (VS)—for their ability to entrap or immobilize lipase and the effects of immobilization on the temperature, pH, operational stability, and reusability of the lipase were investigated. In the present study,* Aspergillus niger* F7-02 lipase was immobilized by entrapment in polysaccharide hydrogel beads of* Irvingia gabonensis* matrix at optimized immobilization conditions for biotechnological industrial application.

## 2. Materials and Methods

### 2.1. Materials

*Irvingia gabonensis* seeds were purchased from a local market in Abeokuta south LGA, Ogun State, Nigeria. It was hot-air oven-dried at 50°C for 2 h. The dried seeds were grounded to powder and defatted in a soxhlet extractor using ethanol as the solvent. The defatted powder was dried at 80°C for 1 h and kept for further studies. All chemicals used were of analytical grade.

### 2.2. Microorganism

*Aspergillus niger* F7-02 was obtained from the culture collection of the Department of Microbiology, Federal University of Agriculture, Abeokuta, maintained on Sabouraud dextrose agar (SDA) at 4°C and subcultured bimonthly.

### 2.3. Chemicals

Glutaraldehyde, formaldehyde, and ethanol were from Sigma Ltd., UK. All chemicals were of reagent grade.

### 2.4. Source of Crude Lipase

*Aspergillus niger* F7-02 was grown on solid-state fermentation medium containing rice bran, palm kernel cake waste, groundnut cake waste, and starch flour in the ratio 5 : 5 : 3 : 1 (%w/w), moistened with 55% water incubated at 30°C for 72 h according to the modified method of Osho et al. [13]. Moldy bran was dissolved in 50 mM sodium phosphate buffer pH 8 (1 : 10 w/v) and the mixture was incubated at 4°C for 3 h with intermittent shaking. The filtered extract was used as a crude enzyme source.

### 2.5. Titrimetric Assay of Lipase Activity

The activity of the enzyme was determined according to the method described by Pinsirodom and Kirk [14] and dos Prazeres et al. [15]. Lipase activity was assayed using olive oil emulsion substrate which was prepared by mixing 25 mL olive oil and 75 mL 7% Arabic gum solution in a rotary shaker at 150 rpm for 5 min. The reaction mixture containing 50 mL olive oil emulsion substrate and 10 mL crude enzyme was incubated at 50°C in an orbital shaking at 160 rpm for 30 min. At five-minute reaction interval (5, 10, 15, 20, and 25 min), 5 mL reaction mixture was removed and each subsample was transferred to a separate 250 mL conical flask containing 10 mL 95% (v/v) ethanol and 3 drops 1% (w/v) thymolphthalein indicator and swirled to stop reaction. The amount of released free fatty acids was titrated with 0.05 N NaOH solution and calculated according to the equation. One unit (U) of lipase activity is the amount of enzyme which liberates from emulsion substrate 1 *μ*mol of fatty acid per mL per minute under specific assay conditions. Consider

(1) μmol  fatty  acid/mL  subsample =mL  NaOH  for  sample−mL  NaOH  for  blank    mL  NaOH  for  sample  −mL  NaOH  for  blank× N×10005 mL−1,

where *N* is the normality of 0.05 NaOH titrant used.

### 2.6. Properties of the Free and Immobilized Lipase

#### 2.6.1. pH Stability

Both free and immobilized enzyme were incubated using citrate buffer (pH 3.0–5.5) and citrate-phosphate buffer (pH 6.0–8.0) at 40°C. After 25 min, the enzyme samples were cooled at 4°C and dialysed against distilled water according to the method of Abdel-Naby et al. [6]. The residual lipase activity was assayed under the standard conditions.

#### 2.6.2. Thermal Stability

The lipase samples were incubated with citrate-phosphate buffer (0.1 mol l^−1^) at the optimum pH value (5.5 for the free enzyme and 4.5 for the immobilized enzyme) at a particular temperature (35–70°C) for 60 min. The residual enzyme activity was assayed under the standard conditions.

#### 2.6.3. Enzyme Kinetics

The kinetic parameters (Km and *V*max apparent) were determined for lipase of* A. niger* F7-02 by measuring enzyme activity at various concentrations (2.0–10.0, v/v) of the olive oil emulsion substrate. The apparent Km and *V*max of the enzyme for the olive oil substrate were determined by plotting the reciprocals of the initial velocities against the reciprocals of the substrate concentrations [16].

#### 2.6.4. Determination of Protein Content of the Crude Lipase

Protein concentration in the crude lipase was determined by biuret method [17]. Enzyme solution (1.0 mL) was mixed with 4 mL biuret reagent and incubated at 37°C for 20 min. The absorbance was measured at 520 nm and protein concentration was determined by extrapolating from the prepared bovine serum albumin (BSA) standard curve.

### 2.7. Partial Purification of Crude Lipase by Imarsil and Activated Charcoal

The crude lipase was partially purified as described by Kareem and Akpan [18]. The enzyme was treated with 1% (w/v) Imarsil and incubated at 4°C for 3 h. The supernatant from treated broth was decanted after which activated charcoal (3% w/v) was added and incubated at 40°C for 1 h. The protein content and also lipase activity of each fraction were assayed as described earlier.

### 2.8. Immobilization of Partially Purified Lipase Using* Irvingia gabonensis*

Adequate gel strength was achieved by dissolving defatted powder of* I. gabonensis* in phosphate buffer pH 7.0 solutions, activated with 2.5% (w/v) glutaraldehyde and thoroughly mixed with enzyme solution. Beads were stabilized by dropping gel through a laboratory dropper into ethanolic-formaldehyde solution and were allowed to stand for 24 h. Beads were dried at 40°C for 3 h. Lipase activity of the immobilized enzyme was assayed as described earlier.

### 2.9. Optimization of Immobilization of Partially Purified Lipase on* I. gabonensis* Matrix

#### 2.9.1. Effect of Enzyme Load on Lipase Activity

Various enzyme concentrations were achieved by dissolving 15.52% (w/v) defatted powder of* I. gabonensis* in phosphate buffer solution (pH 7.0) containing 15, 25, 35, 45, and 60% (v/v) enzyme solution activated with 2.5% (v/v) glutaraldehyde solution. Beads were formed and activity was assayed as described earlier.

#### 2.9.2. Effect of Bead Size on Lipase Activity

Optimum enzyme concentration was used to prepare the gel. Various bead sizes (5–9 mm) were achieved by dropping gel through laboratory dropper of various diameter sizes. Lipase activity was assayed as described earlier.

#### 2.9.3. Effect of Number of Beads on Lipase Activity

At optimum enzyme load and bead size, various numbers of beads (5–25) were used to hydrolyze lipids to study the effect on lipase activity. Lipase activity was assayed as described earlier.

#### 2.9.4. Effect of Beads Reusability on Lipase Activity

Optimum number of beads was used in a repeated batch cycle to study the effect of reusability on lipase activity. At the end of each batch, the beads were washed in phosphate buffer (pH 7.0), dried, and used for the next batch. The lipase activity at the end of each batch was assayed as described earlier.

### 2.10. Activity of Immobilized Lipase on* I. gabonensis* Matrix

Dizge et al. [19] procedure was adopted in which free and immobilized lipases activities were determined using the optimum conditions obtained to evaluate the activity yield of the immobilized lipase on* I. gabonensis* matrix by the formula below:

(2) Lipase  activity(U/g-support)  =activity  of  immobilized  lipaseamount  of  support  used,Specific  activity(U/mg  protein)  =activity  of  immobilized  lipaseamount  of  protein  loaded,Activity  yield(%)  =specific  activity  of  immobilized  lipasespecific  activity  of  free  lipase×100%.

## 3. Results and Discussion

### 3.1. Immobilization of Partially Purified Lipase Using* I. gabonensis*

**Figure 1:**
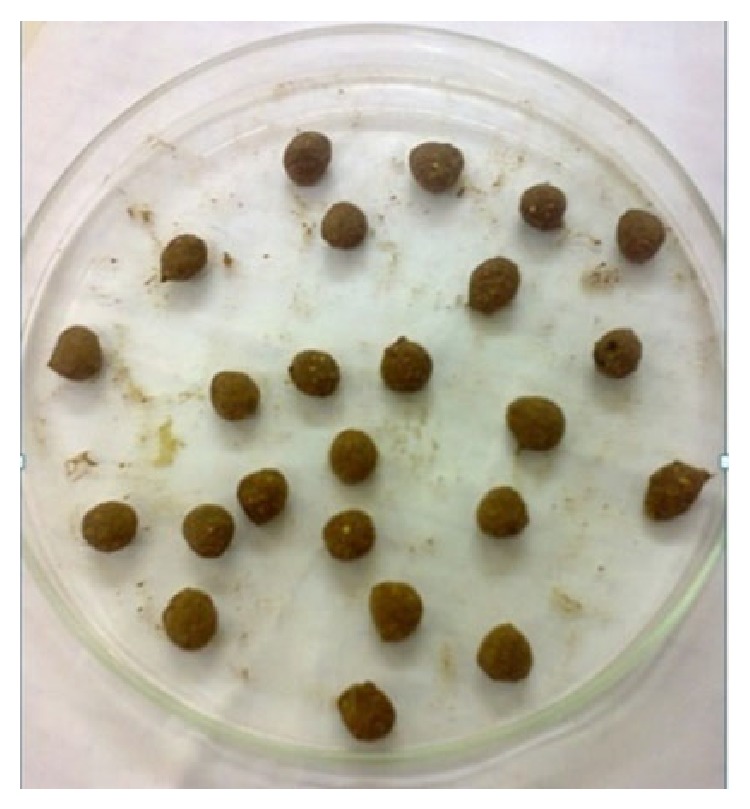
Immobilized lipase of* A. niger* F7-02 in hydrogel beads of* Irvingia gabonensis*.

Adequate gel strength was achieved by dissolving defatted powder of* I. gabonensis* 15.52% (w/v) in phosphate buffer (pH 7.0) solution, activated with glutaraldehyde 2.5% (w/v) and thoroughly mixed with partially purified lipase 15% (v/v) solution. Glutaraldehyde provides a powerful covalent link between the microbial cell and its carrier matrix as the beads were stabilized in ethanolic-formaldehyde 3 : 1 (v/v) solution for 24 h.  Figure 1 shows the immobilized lipase of* A. niger* F7-02 in hydrogel beads of* I. gabonensis.* Solidification with formaldehyde enhances stability of the matrix and eventually overcomes the tendency to agglomerate or form a gel in aqueous solutions but higher formaldehyde concentration imposed diffusional limitations by the solid nature of the hardened matrix [20–22].

At low concentration of the defatted powder, the beads were unstable and fragile which led to poor immobilization. This may be due to the larger pore size in the gel which probably caused leaching of enzyme. At concentration above 15.52% (w/v), the gel was too hard and could not split out of the dropper which may result in stearic hindrance of enzyme due to high ethanol concentration with harder beads formed [23]. Stable beads were achieved by dropping in ethanolic-formaldehyde 3 : 1 (v/v) solution while further increase in ethanol concentration with decrease of formaldehyde concentration though produced stable beads, but with decrease activity, with no activity at 9 : 1 (v/v) concentration. However, reducing ethanol concentration with increasing formaldehyde concentration results in unstable beads formation, suggesting ethanol as a key hardening agent. This supports the finding of Osho et al. [22] that obtained 3 : 1 (v/v) as optimal ethanolic-formaldehyde solution for bead stability in the production of amylase.

### 3.2. Partial Purification of Crude Lipase by Imarsil and Activated Charcoal

The partial purification of the crude enzyme by Imarsil and activated charcoal led to the reduction in protein content from 4.44 mgml^−1^ to 3.65 mgml^−1^ which consequently increased the lipase activity from 23.5 Uml^−1^ to 76.2 Uml^−1^ (Table 1). There are various methods for concentrating dilute enzyme solutions and proteins from fermentation broth or cell extracts using coagulants from agricultural materials. Kareem et al. [24] reported* Calotropis procera* (Sodom apple) as a potential material for enzyme purification. This novel and inexpensive synthetic chromatographic absorbent, Imarsil, has also been used by Kareem and Akpan [18] for clarification of amylase extract from moldy bran. However, in this study Imarsil and activated charcoal were used to purify crude lipase in a two-step purification fold, resulting in an increase in specific activity from 5.29 to 20.8 Umg^−1^ proteins with 3.93-fold purification and 18.24% protein reduction in the supernatant. This study has further validated the use of Imarsil as a coagulating-flocculating agent in purification of enzyme extracts.

### 3.3. Properties of the Free and Immobilized Lipase of* A. niger* F7-02

#### 3.3.1. pH Stability

**Figure 2:**
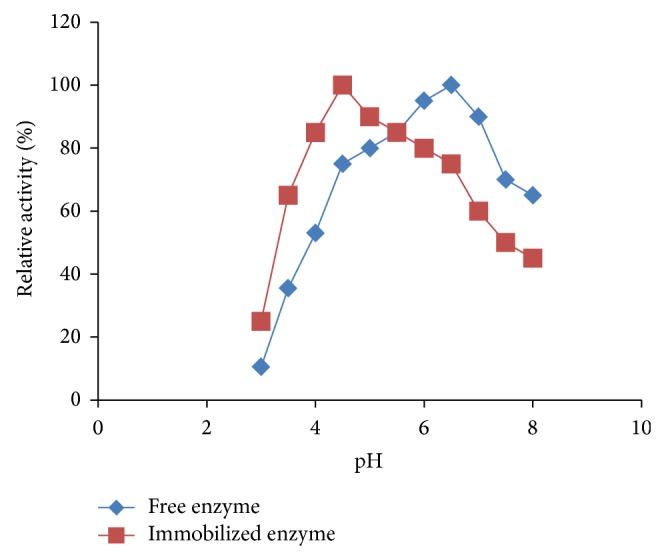
pH stability of free and immobilized lipase of* A. niger* F7-02.

The pH dependence of the activity of free and immobilized lipase was obtained in 0.1 mol l^−1^ citrate-phosphate buffer at 45°C for the catalytic activity. The optimum pH of the free lipase was pH 6.5 while that of immobilized enzyme tends towards acidic medium with optimum pH 4.5 (Figure 2). This may be a result of the microenvironmental pH of the polysaccharide hydrogel support. The works of Abdel-Naby et al. [6] and Krajewska et al. [25] confirmed the finding that the positively charged supports displace pH-activity curves of the lipase attached to them towards lower pH values.

#### 3.3.2. Thermal Stability

**Figure 3:**
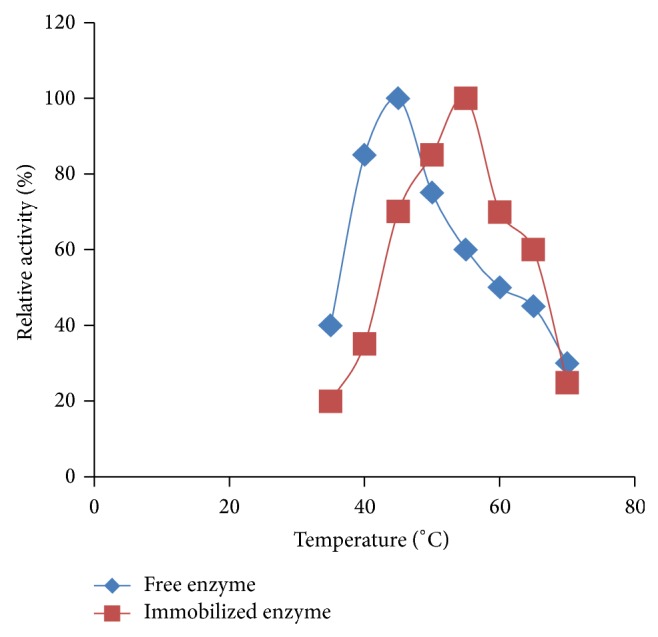
Thermal stability of free and immobilized lipase of* A. niger* F7-02.

The optimum temperature of the free and immobilized enzyme was obtained in the buffer with the optimum pH (pH 6.5 and 4.5, resp.). The optimum temperature of the free lipase was about 45°C, whereas that of the immobilized enzyme was increased to 55°C (Figure 3). Osho et al. [12] reported in their study on the effect of reaction temperature on lipase activity using structural fibrous network of papaya and vegetable sponge-immobilized lipases of* A. niger* ATCC 1015 recorded optimum temperature of 45°C for both matrices and ascribed the reason to a consequence of enhanced thermal stability. In support of this finding, Allenza et al. [26] and Kitano et al. [27] reported that the activation energy of the immobilized enzyme was lower than that of the free enzyme (i.e., the higher the temperature, the lower the activation energy), because the internal diffusion of the substrate into the carrier-enzyme system was the rate-limiting the process.

#### 3.3.3. Kinetic Assessment of the Enzyme Hydrolysis

**Figure 4:**
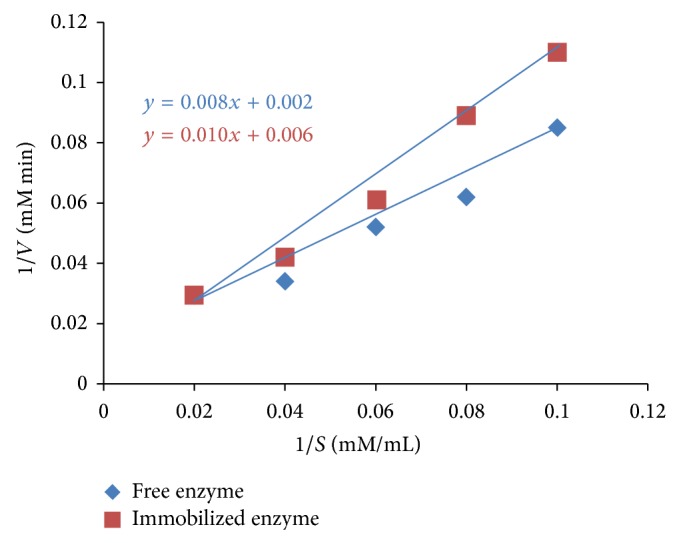
Kinetic assessments of* A. niger* F7-02 free enzyme (slope = 0.008, 1/*V*max = 0.002, Km = slope/1/*V*max, and Km 0.008/0.002 = 4.0 mM) and immobilized enzyme (slope = 0.01, 1/*V*max = 0.006, Km = slope/1/*V*max, and Km 0.01/0.006 = 1.7 mM).

The result of kinetic assessments of enzyme hydrolysis of free lipase from* A. niger* F7-02 (Figure 4) showed a Km value of 4.0 mM and *V*max 2.0 × 10^−3^ millimole min^−1^ and the immobilized lipase revealed a Km value of 1.7 mM and *V*max 6.0 × 10^−3^. The Michaelis constant (Km) indicated 2.35-fold affinity of the immobilized lipase produced by* A. niger* F7-02 towards the substrate as compared to the free lipase.

### 3.4. Optimization Studies of* I. gabonensis* Immobilized Lipase 

#### 3.4.1. Effect of Enzyme Load on Lipase Activity

**Figure 5:**
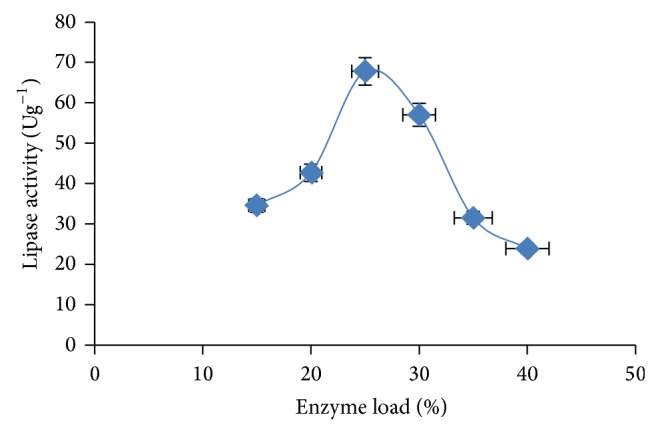
Effect of enzyme load on lipase activity. The error bars with 5% value of three samples.

The influence of initial enzyme load was optimum at 25% (v/v) concentration with activity of 67.8 Ug^−1^ (Figure 5). Higher lipase concentration resulted into decrease activity and this may be due to decrease in mechanical strength gel particles with increasing enzyme concentration as reported by Osho et al. [22]. Shinmyo et al. [28] have also shown that at higher gel concentrations the rate of substrate (starch) mass transfer and the enzyme yield decreases.

#### 3.4.2. Effect of Bead Size on Lipase Activity

**Figure 6:**
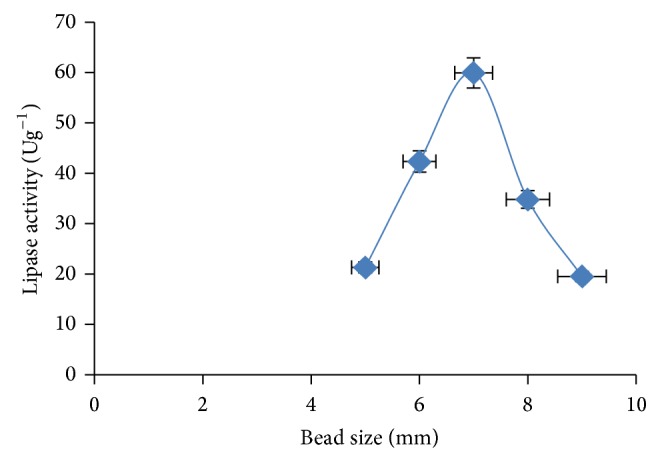
Effect of bead size on lipase activity. The error bars with 5% value of three samples.

Bead size has significant effect on lipase activity. Optimum activity (59.9 Ug^−1^) was achieved at 7 mm size and decreased with further increase in bead size (Figure 6). At low bead size, the gel matrix is thinner and as such lipase has more access to substrate.

#### 3.4.3. Effect of Number of Beads on Lipase Activity

**Figure 7:**
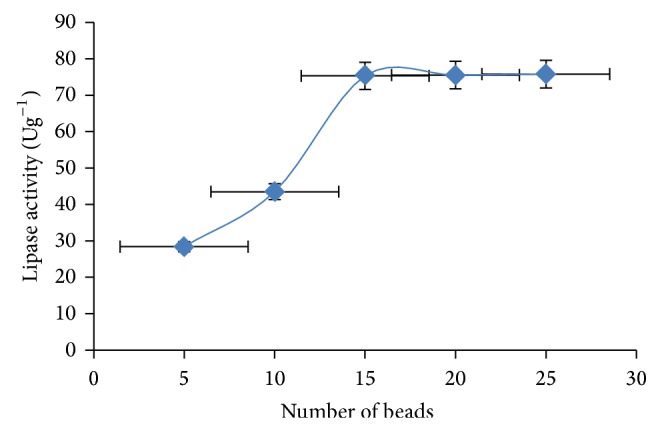
Effect of number of beads on lipase activity. The error bars with 5% value of three samples.

For the effect of number of beads on activity, lipase activity increases as the number of beads increases and achieved 75.3 Ug^−1^ optimum activities with 15 beads (Figure 7). However, with further increase in bead number, activity became asymptotic. This agrees with Vilmaminovska and Slobodankakuzmanova [24], as reaction rate was maximum at 15 beads, so further increase in beads number will achieve the same rate.

#### 3.4.4. Effect of Bead Reusability on Lipase Activity

**Figure 8:**
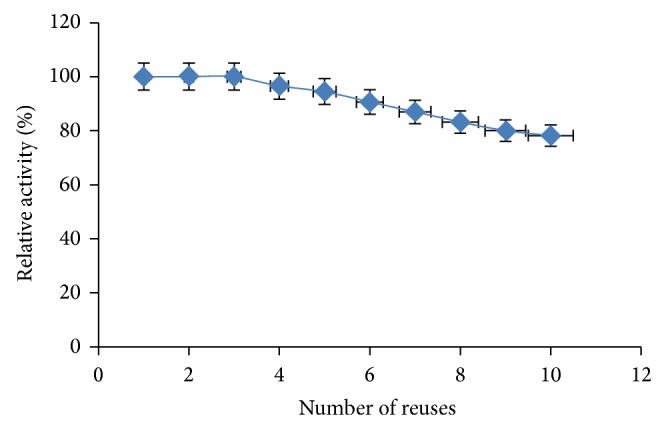
Effect of beads reusability on lipase activity. The error bars with 5% value of three samples.

The relative activity on reusability of the* I. gabonensis* immobilized lipase is presented in Figure 8. Maximum lipase activity obtained was 78.5 Ug^−1^ (not shown) and 80% relative activity was retained at 8 repeated batch cycles. Thereafter, there was sharp decline in the activity of the immobilized enzyme. The reduction in activity can be attributed to weakening of binding strength between the support and the enzyme on repeated use and, hence, enzyme leached out from the matrix. This is in agreement with the reports of Jaiswal and Prakash [5] and Osho et al. [22].

### 3.5. Activity Yield of Immobilized Lipase on* I. gabonensis* Matrix

The immobilization process gave activity yield of 59.1% with specific activity of 12.3 Umg^−1^ and lipase activity of 67.8 Ug^−1^ supports as shown in Table 2. These results are higher than that of Dizge et al. [19]; they reported activity yield of 6.10 Ug^−1^ with lipase immobilized on styrene-divinylbenzene (STY-DVB) copolymer. The effectiveness of* I. gabonensis* as a polymer matrix for lipase immobilization has thus been established and it is cost-efficient as compared to synthetic polymer often used. There was an increase in protein concentration of the immobilized lipase compared to that of the free lipase. This consequently enhanced the activity yield of the immobilized enzyme as it affects the enzyme activity.

## 4. Concluding Remarks

This study has presented the potential of* I. gabonensis* matrix as a suitable matrix for the immobilization of* A. niger* F7-02 lipase. Immobilized enzyme was found stable and can retain about 80% of initial yield after eight repeated uses. It is an abundantly available and environment-friendly material which can promote large scale and economical production of commercially valuable organic acid.

## Figures and Tables

**Table 1:** Partial purification of crude lipase of *A. niger* F7-02.

Sample	Lipase activity (Ug^−1^)	Protein concentration (mgmL^−1^)	Specific activity (Umg^−1^)	Purification fold
Crude enzyme	23.5	4.44	5.29	—
Partially purified enzyme	76.2	3.65	20.8	3.93

**Table 2:** Activity yield of immobilized lipase on *I. gabonensis* matrix.

Parameter	Free enzyme	Immobilized enzyme
Lipase activity (Ug^−1^)	76.2	67.8
Protein concentration (mgmL^−1^)	3.65	5.5
Specific activity (Umg^−1^)	20.8	12.3
Activity yield (%)	—	59.13
